# Expanding the genotype–phenotype correlation of childhood sensory polyneuropathy of genetic origin

**DOI:** 10.1038/s41598-020-73219-5

**Published:** 2020-09-30

**Authors:** Samya Chakravorty, Rachel Logan, Molly J. Elson, Rebecca R. Luke, Sumit Verma

**Affiliations:** 1 Department of Pediatrics, Emory University School of Medicine, 2015 Uppergate Drive, Atlanta, GA 30322 USA; 2 Neurosciences Research, Children’s Healthcare of Atlanta, Georgia, USA; 3 Department of Human Genetics, Emory University School of Medicine, Atlanta, GA USA; 4 School of Biological Sciences, Georgia Institute of Technology, Atlanta, GA USA; 5 Emory University School of Medicine, Atlanta, GA USA; 6 Cook Children’s Hospital, Fort Worth, TX USA; 7 Department of Pediatrics and Neurology, Emory University School of Medicine, 1400 Tullie Road, 8th Floor, Atlanta, GA 30329 USA

**Keywords:** Clinical genetics, Medical genetics, Neuropathic pain, Paediatric neurological disorders, Peripheral neuropathies, Spinocerebellar ataxia

## Abstract

Pure sensory polyneuropathy of genetic origin is rare in childhood and hence important to document the clinical and genetic etiologies from single or multi-center studies. This study focuses on a retrospective chart-review of neurological examinations and genetic and electrodiagnostic data of confirmed sensory polyneuropathy in subjects at a tertiary-care Children’s Hospital from 2013 to 2019. Twenty subjects were identified and included. Neurological examination and electrodiagnostic testing showed gait-difficulties, absent tendon reflexes, decreased joint-position, positive Romberg’s test and large fiber sensory polyneuropathy on sensory nerve conduction studies in all patients associated with lower-extremity spasticity (6), cardiac abnormalities or cardiomyopathy (5), developmental delay (4), scoliosis (3), epilepsy (3) and hearing-difficulties (2). Confirmation of genetic diagnosis in correlation with clinical presentation was obtained in all cases (*COX20* n = 2, *HADHA* n = 2, *POLG* n = 1, *FXN* n = 4, *ATXN2* n = 3, *ATM* n = 3, *GAN* n = 2, *SPG7* n = 1, *ZFYVE26* n = 1, *FH* n = 1). Our single-center study shows genetic sensory polyneuropathies associated with progressive neurodegenerative disorders such as mitochondrial ataxia, Friedreich ataxia, spinocerebellar ataxia type 2, ataxia telangiectasia, spastic paraplegia, giant axonal neuropathy, and fumarate hydratase deficiency. We also present our cohort data in light of clinical features reported for each gene-specific disease subtype in the literature and highlight the importance of genetic testing in the relevant clinical context of electrophysiological findings of peripheral sensory polyneuropathy.

## Introduction

Sensory polyneuropathy is rare in children^1^ with a broad differential diagnosis that includes both acquired and genetic etiologies^2^. Exposure to medications or supplements, including multiple chemotherapy agents such as cisplatin, carboplatin, and pyridoxine may induce a large fiber predominant sensory polyneuropathy. Trauma, compression, injury during surgical procedures, infections (Leprosy, Human Immunodeficiency Virus), vitamin B12 deficiency, diabetes mellitus and the rare Miller Fisher variant of Guillain–Barré syndrome (GBS) also present with definitive sensory dysfunction^3^. Genetic etiologies cause a heterogeneous group of progressive neurodegenerative disorders, but a paucity of literature on detailed clinical presentation, electrodiagnostic, and genetic-workup for children’s sensory polyneuropathy necessitates further characterization. In this study, we describe a single center pediatric electrodiagnostic (EDX) laboratory experience identifying pure sensory polyneuropathy on nerve conduction study (NCS) with detailed clinical features and genetic etiologies and compared each subtype findings to available English literature on rare inherited sensory polyneuropathies.

## Results

Twelve hundred sixty one pediatric EDX studies were performed from 2013 to 2019. One hundred fifty seven studies (12%) were classified as sensory motor polyneuropathy and 24 studies (2%) had pure sensory polyneuropathy without gender preference. Further review of the subjects with pure sensory polyneuropathy on NCS revealed 20 children (12 girls, 8 boys, mean age of 13.6 ± 4.1 years) with confirmed genetic diagnosis with clinical correlation. The remaining 4 children had acquired sensory polyneuropathy i.e. Miller Fisher variant GBS, diabetes mellitus and were excluded from this study.

The study cohort presented with gait difficulties and on examination had sensory ataxia 100% (n = 20), spasticity 30% (n = 6), global developmental delay 20% (n = 4), cardiac defects or cardiomyopathy 25% (n = 5), scoliosis 15% (n = 3), epilepsy 15% (n = 3), and hearing difficulties 10% (n = 2). Significant family histories of neuromuscular illness or parental consanguinity were absent. Study subjects had genetic confirmation with pathogenic variants in mitochondrial genes [30%: *COX20* (OMIM#614698) n = 2, *HADHA* (OMIM#600890) n = 2, *POLG* (OMIM#174763) n = 1, *FH* (OMIM# 136850) n = 1], Friedreich-ataxia (21%: *FXN* (OMIM#606829) n = 4), spinocerebellar ataxia type-2 (15%: *ATXN2* (OMIM#601517) n = 3), ataxia-telangiectasia (15%: *ATM* (OMIM#607585) n = 3), giant axonal neuropathy (10%: *GAN* (OMIM# 605379), n = 2), and 5% each in spastic paraplegia 7 (*SPG7* (OMIM#602783) n = 1) and spastic paraplegia 15 (*ZFYVE26* (OMIM# 612012) n = 1). Subjects on an average had a 4.9 ± 3.3 years delay from symptom-onset to genetic confirmation. Clinical presentation with genetic variant and review of literature are presented in Tables 1 and 2. Below we give detailed genotypic and phenotypic features of subtypes of genetic childhood polyneuropathies identified in this study.

**Table 1:** Patients with sensory polyneuropathy of genetic etiology.

Patient#	Age (years/weeks) at presentation (current age in years)	Sex	Gene	Variant(s)	Clinical presentation of the cohort
**Mitochondrial sensory polyneuropathy**					
1**	1 year (20)	F	*POLG*	[c.2243G>C (p.W784S)]; [c.3609_3612dupAACT]	Epilepsy, ataxia, speech delays, good strength on exam, nystagmus, dysarthria, and absent reflexes, liver problems
2	10 years (14)	F	*HADHA*	[c.1528G>C, (p.E510Q)]; [c.1620 + 2_162 + 0delTAAGG]	Retinitis pigmentosa, cardiac atrioventricular (AV) valve insufficiency, seizures with episodic weakness and gait abnormalities. Tightened heel cords, decreased lower extremity reflexes, difficulty walking on heels and normal strength on exam
3	1 year (5)	M	*HADHA*	[c.1418C>A, (p.Ala473Asp)]	Cardiomyopathy, difficulty walking, and motor delay; increased heel cords, slightly decreased strength and reflexes in lower extremities, bilateral hand tremors, and waddling gait on exam
4*	4 years (10)	M	*COX20*	[c.41A>G (p.K14R)]; [c.157 + 3G>C]; [c.340G>A (p.Gly114Ser)]	Respiratory distress, speech and gross motor delays, alternating esotropia, strabismus, gait ataxia, hypotonia and hyperreflexia, sensory polyneuropathy per EMG/NCS
5*	4 years (18)	F	*COX20*	[c.41A>G (p.K14R)]; [c.157 + 3G>C]; [c.340G>A (p.Gly114Ser)]	distal-extremity weakness, spasticity, and hearing loss, farsightedness and esotropia, developed gait instability, chronic gastrointestinal problems with family history of Celiac disease
6	10 years (11)	M	*FH*	[c.697C>T (p.R233C)]; [c.1431_1433dup]	Focal epilepsy seizures and infantile spasms, abnormal MRI, strabismus and visual impairment, fumarate hydratase deficiency, weight gain, fatigues, weakness, generalized severe hypotonia, global developmental delay, anemia, focal cerebral dysfunction, cardiac defects, pulmonary artery hypertension, pulmonary stenosis, urinary tract infection, and vomiting
7*	3 weeks (17)	M	*ATXN2*	[c.1564_1565delGA (p.Glu522Ilefs43)]	Ataxia, developmental delay, and myoclonic jerks (onset age 10). Myoclonic jerks, bradykinesia, spasticity, decreased pinprick, vibration and proprioception in his feet, dysmetria, absent reflexes in low extremities and wide based ataxic gait on exam
8*	3 weeks (17)	M	*ATXN2*		Ataxia, developmental delay, and myoclonic jerks
9	4 years (14)	F	*ATXN2*	64 and 22 CAG repeats	Ataxia, dysphagia, developmental delay, dysarthria, increased tone in extremities and axial hypotonia, decreased vibration and proprioception, ataxia and dysmetria, and absent tendon reflexes
**Ataxia telangiectasia**					
10*	6 years (12)	F	*ATM*	[c.1564_1565delGA (p.Glu522Ilefs43)]	Ataxia, telangiectasia and myoclonus
11*	4 years (11)	F	*ATM*	[c.1564_1565delGA (p.Glu522Ilefs43)]	Myoclonus, ataxia has been progressing and has been clumsy since infancy. Decreased reflexes, nystagmus and dysarthria on exam
12	3 years	F	*ATM*	[c.4544dup (p.Asn1515Lysfs*16)]; [c.7397C>A (p.Ala2466Glu)]; [c.7502A>G (p.Asn2501Ser)]	Balance problems, drooling and fine motor problems, in-toeing of gait, frequent ear infections, difficulty maintaining sleep, suggestive of sensory ataxia
13	9 years (10)	F	*FXN*	933 & 933 GAA triplet repeats	Gait difficulty since early childhood , pain in her legs, very poorly coordinated
14	6 years (16)	F	*FXN*	1250 & 899 GAA repeats	Ataxia, developmental delay, progressive ataxia. Good strength but decreased vibration in her extremities with ataxia and present reflexes on exam
15	3 years (10)	M	*FXN*	933 & 10 GAA repeats; [c.317 T>C, (p.Leu106Ser)]	Ataxia, leg pain, easy fatigue, attention deficit hyperactivity disorder (ADHD)
16	8 years (10)	F	*FXN*	1066 and 866 GAA repeats	Cardiomyopathy, poor coordination, abnormal gait, scoliosis, bladder dysfunction, and ataxia
**Spastic paraplegia**					
17	4 years (16)	F	*ZFYVE26*	[c.2300G>A, (p.767H)]; [c.2799C>T, (p.L933 =)]	Progressive ataxia, seizures, scoliosis, cerebellar atrophy, and hearing loss. Hypotonia, absent reflexes in lower extremities, ataxia, and tremulousness on exam
18	10 years (18)	M	*SPG7*	[c.1A>G, p.M?]	Scoliosis and gait abnormalities. Decreased strength in lower extremities with contractures, decreased vibration and temperature sensation, and diminished reflexes in lower extremities on exam
**Giant axonal neuropathy**					
19	6 years (10)	M	*GAN*	[c.851 + 1G>A] (homozygous)	Progressive gait abnormality, hammer toes, high arched feet bilaterally, absent distal tendon reflexes in the lower extremities, abnormal brain MRI
20	2 years (10)	F	*GAN*	[c.805C>T (p.Arg269Trp)]; [c.732delT (p.Ile244MetfsX33)]	Gait abnormality, decreased muscle bulk in the bilateral lower extremities and distal hands, absent deep tendon reflexes, flexor plantar responses, vocal cord paralysis, episodes of tachycardia, difficulty breathing, poor cough reflex, difficulty in swallowing

*M* male, *F* female.

*Siblings, **Deceased.

**Table 2:** Review of literature for each genetic sensory polyneuropathy subtype identified.

Genetic sensory polyneuropathy subtypes	Gene involved	Clinical presentations published in the English literature survey that correlated with each gene-specific subtype
Mitochondrial sensory polyneuropathy	*POLG*	(a) Lactic acidosis, seizures, ataxia, peripheral neuropathy, developmental delay, myopathy, chronic progressive external ophthalmoplegia, or hepatopathy^5^; (b) Autosomal dominant form of progressive external ophthalmoplegia (PEO) with mitochondrial DNA deletions^6–8^; (c) Autosomal recessive form of sensory ataxic neuropathy, dysarthria, ophthalmoparesis (SANDO)^9^; (d) Spinocerebellar ataxia with epilepsy (SCAE)^10^; Five patients with PEO, but one patient with previously-reported *POLG* polymorphism but likely a damaging variant showed only mild distal muscle atrophy without ophthalmoplegia^11^; (e) Mitochondrial DNA Depletion Syndrome 4A (Alpers Type), Alpers–Huttenlocher syndrome (AHS or Leigh Syndrome) including spastic diplegia due to anoxic encephalopathy, clinical triad of psychomotor retardation, intractable epilepsy, and liver failure^12–16^; (f) Myocerebrohepatopathy spectrum (MCHS) mitochondrial neurogastrointestinal encephalopathy syndrome (MNGIE)^9,17^; (g) MNGIE with severe hypotonia, gastrointestinal dysmotility, and mtDNA depletion in muscle^18^; Charcot‐Marie‐Toothdisease^19^
	*HADHA*	(a) Long-chain 3-hydroxyl-CoA dehydrogenase deficiency (LCHADD) with hypoketotic hypoglycemia and fatty liver in children^20^; (b) Mitochondrial Trifunctional Protein (MTP) Deficiency: hypoglycemia, cardiomyopathy and sudden death at the age of 18 months^21^; (c) Children with complete MTP deficiency with neonatal dilated cardiomyopathy or progressive neuromyopathy^20^; (d) LCHAD deficiency in 3 families where children had sudden death or hypoglycemia and abnormal liver enzymes (Reye-like syndrome)^22^; (e) Recurrent rhabdomyolyses, myoglobinuria, neuropathy, distal muscle atrophy, exercice intolerance, Neuropathy (axonal, sensorimotor), distal muscle atrophy, elevated creatine kinase^23^; (f) Lethal types: hypotonia, coma, cardiomyopathy, cardiac failure^24,25^; (g) Acute metabolic problems with infections, rhabdomyolysis, peripapillary chorioretinal atrophy, diffuse granular appearance of the macular retinal pigment epithelium^26^
	*COX20*	(a) Growth retardation, hypotonia, and cerebellar ataxia^27^; (b) 2 siblings showing combination of childhood-onset cerebellar ataxia, dystonia, and sensory axonal neuropathy, no cognitive defects, different from typical respiratory chain disorders^28^; (c) Four subjects with childhood hypotonia, areflexia, ataxia, dysarthria, dystonia, and sensory neuropathy^29^; (d) Sensory-dominant axonal neuropathy, static encephalopathy, mild muscle weakness of bilateral legs, dysesthesia, dysarthria and intellectual disability, no cerebellar abnormality^30^
	*FH*	(a) Early-onset hypotonia, psychomotor retardation, brain abnormalities including corpus callosum agenesis, gyral defects, and ventriculomegaly, sometimes neonatal distress, metabolic acidosis, encephalopathy^31,32^; (b) Two siblings with progressive encephalopathy, dystonia, leucopenia, and neutropenia, lactate elevation in cerebrospinal fluid, high fumarate excretion in urine, tricarboxylic acid cycle and fumarase deficiency^33^; (c) Male infant with mitochondrial encephalopathy, onset age 1 month, failure to thrive, developmental delay, hypotonia, cerebral atrophy, lactic and pyruvic acidemia, fumaric aciduria, glutamate and succinate oxidation defects in skeletal muscle mitochondria, death at 8 months age^34^; (d) Mental retardation, onset age 6 months, hypotonia, microcephaly, delayed development^35^; mild hyperammonemia in fumarase deficiency^36^; (e) Childhood dementia, generalized seizures, psychomotor deterioration, fumaric aciduria, death at 7 month age^37^; (f) Patients (20 months-12yrs age) in a large consanguineous US family: developmentally retarded, no language development, most are unable to sit/walk, macrocephaly, ventricular enlargement, polymicrogyria, frontal horns angulation, decreased periventricular white matter, small brainstem hypotonia, seizures, status epilepticus, frontal bossing, hypertelorism, depressed nasal bridge, anteverted nares, high-arched palate, most have polycythemia at birth, some have optic nerve hypoplasia or pallor^31^; (g) Two brothers: hypotonia, respiratory insufficiency after birth, corpus callosum agenesis, ventriculomegaly, dangling choroid plexus, bilateral renal pyelectasis, ventriculoseptal defect, death at 22 days age, postmortem showed lissencephaly, severe metabolic acidosis, necrotizing enterocolitis, liver failure with coagulopathy, hyperbilirubinemia, and encephalopathy^32^; non-inflammatory biliary atresia^38^; (h) 2.5 year old girl with significant constipation, developmental regression with time, facial dysmorphism with microcephaly, bilateral epicanthal folds, downward palpebral fissures, strabismus, slight tapering of fingers, mild fifth finger clinodactyly. global hypotonia, mild ataxic gait, cerebral atrophy, thin corpus callosum, unusual behaviors including self-injurious behaviors^39^; (i) Clinical heterogeneity: 1st patient with severe neonatal encephalopathy, polymicrogyria, < 1% fumarase activity, 2nd patient had microcephaly, mental retardation, 20% fumarase activity, 3rd patient had mild mental retardation, polymicrogyria, 42–61% fumarase activity^40^; (j) Boy with developmental and growth delay, microcephaly, hypotonia, age of onset 3 months^41^; (k) 8 year old girl with milder symptoms: hypotonic since birth, short apneic crises, leg and arm spasms, grand mal seizures, facial dysmorphism with depressed nasal bridge, anteverted ears, hypertelorism, microcephaly, speech defects, spastic paraparesis, brain MRI showed slight ventriculomegaly, white-matter atrophy and corpus callosum hypoplasia^42^; (l) Early infantile encephalopathy with severe developmental retardation, hypotonia, seizures, brain malformations, diffuse polymicrogyria, decreased cerebral white matter, large ventricles, open opercula, dysmorphic facial features, neonatal polycythemia^31^; (m) Sibling boys with polyhydramnios and enlarged cerebral ventricles in utero, and subsequently cerebral atrophy, severe developmental delay, infantile spasms^43^
Spinocerebellar ataxia (SCA)	*ATXN2*	(a) Spinocerebellar Ataxia 2, myoclonus, dystonia, and myokymia increased with number of CAG repeats, susceptibility to later-onset amyotrophic lateral sclerosis 13 (ALS)^44–46^; (b) Autosomal dominant ATXN2>35 CAG repeats causing Spinocerebellar Ataxia 2^47^; (c) Severe early onset obesity in children due to ATXN2 polymorphisms^48^
Ataxia Telangiectasia	*ATM*	(a) Diverse phenotype that includes progressive cerebellar ataxia, oculocutaneous telangiectasias, radiation hypersensitivity, increased cancer incidence, immunodeficiency, chromosome instability, and elevated levels of serum alpha-fetoprotein^49^; (b) Progressive cerebellar ataxia, resulting in wheelchair-bound by teenage, speech-difficulties and abnormal eye movements^50–52^; (c) Increased predisposition to leukemias, lymphomas, and breast cancer^53^
Friedreich's ataxia	*FXN*	(a) Neurodegenerative disorder characterized by progressive limb and gait ataxia, absent lower-limb reflexes, impaired posterior column sensory modalities and up-going plantar responses and possibly complicated by cardiomyopathy, diabetes mellitus, carbohydrate intolerance, and a reduced insulin response to arginine stimulation^54–57^; (b) None to moderate ataxic gait, none to very mild dysarthria, normal to brisk reflexes, no cardiac problem to cardiomyopathy, no diabetes, sensory neuropathy, pas cavus, none to mild scoliosis^58^; (c) Classic early-onset Friedreich ataxia (FA)^59,60^; Mean allele repeat length significantly higher in FA patients with diabetes and those with cardiomyopathy and loss of reflexes in upper limbs^61^; (d) Atypical Friedreich ataxia with slightly later onset had intact tendon reflexes^62,63^; Atypical FAs: later-onset FA (LOFA), FA with retained reflexes (FARR) with or without cardiomyopathy^64^; (e) Identified association between size of the smaller of the 2 expanded alleles and age at onset, age into wheelchair, scoliosis, impaired vibration sense, and the presence of foot deformity, larger allele size associated with bladder symptoms and presence of foot deformity^65^; (f) Developmental delay, hypotonia, decreased stamina, clumsiness, and balance difficulties, mild concentric left ventricular hypertrophy, diastolic dysfunction, thickened ventricular septum (Z score>9), scoliosis of 14°, ultimately wheel-chaired due to scoliosis^66^; (g) Milder atypical FA caused by *FXN* p.R165P variant carriers became wheelchair bound early, retaining reflexes, better arm function, milder dysarthria^67^; Some FXN missense variants cause lower severity, milder phenotypes of FA^68^
Spastic paraplegia	*ZFYVE26*	(a) Thin corpus callosum and white matter abnormalities, motor neuropathy^69^; (b) Pigmentary maculopathy, cerebellar signs and dystal amyotrophy^70,71^; Gait abnormality, mental retardation and learning difficulties, thin corpus callosum and/or white matter lesions abnormalities, mild signs of retinal degeneration in one case, bradykinesia and rigidity at upper limbs, axonal motor neuropathy, chronic neurogenic alterations^72^; (c) Severe lower limb (LL) spasticity, very brisk LL reflexes, severe LL weakness, LL amyotrophy, Babinski sign, mild to moderate upper limb (UL) spasticity, very brisk UL reflexes, mild to severe UL weakness, UL amyotrophy, dysarthria, none to decreased vibration sense, none to some urinary symptoms, mental retardation or deterioration, pigmentary retinopathy, frontotemporal dementia, nystagmus, pseudobulbar signs, epilepsy, hand tremors, diabetes, behavioral disturbances, limited lateral oculomotricity, cerebral, cortical, and cerebellar atrophy, axonal peripheral polyneuropathy^73^
	*SPG7*	(a) Asperger's symptoms and ADHD, slow saccades, moderate carpal tunnel^69^; (b) Cerebellar ataxia with spasticity and waddling gait^74^; (c) Urinary urgency, nystagmus, dysarthria, spasticity, hyper-reflexia, ataxia dysmetria, cerebellar atrophy^75^
Giant axonal neuropathy	*GAN*	(a) Peripheral and central nervous system abnormalities, pale, tightly curled hair, delayed motor milestones and intellectual ability, wheel-chaired at 8 yrs, up-beat nystagmus, slight bilateral facial weakness, and slight atrophy of the tongue, severely ataxic speech, arm movements, and gait, distal paresis, rebound phenomena, absent tendon reflexes, lumbar scoliosis^76^; (b) Hypotonia and clumsy gait, signs of pyramidal tract and cerebellar involvement, motor and cognitive deterioration, wheelchair-bound, severe bladder incontinence, foot deformities, decreased sensory and motor nerve conduction velocities^77^; (c) Atypical giant axonal neuropathy: Charcot–Marie–Tooth 2-like phenotype with foot deformity, distal amyotrophy of lower limbs, areflexia, distal lower limb hypoesthesia, mild cerebellar dysarthria and nystagmus, cerebellar atrophy^78^; (d) Variable phenotypes, classic GAN with kinky red hair, cerebellar ataxia, and peripheral motor and sensory neuropathy, another patient with frizzy hair, spastic paraparesis with Babinski sign, facial diplegia, and minor clinical signs of neuropathy and cerebellar ataxia, another patient had a congenital neuropathy with mental retardation and a rapid and severe progression, but without abnormal hair, another patient had onset at age 3 years of weakness of face, distal and proximal limbs, short stature, foot and hand deformities, scoliosis, sensory impairment^79^; (e) Delayed motor development, unstable gait with areflexia, large head and frizzy hair, brain MRI showed relatively large lateral ventricles, mild cognitive delay^80^; (f) Progressive gait difficulties, weakness and muscular atrophy, extremely curly hair, slightly left palpebral ptosis and horizontal nystagmus, winged scapula and lumbar hyperlordosis, flaccid paraparesis, areflexia, and ataxic gait in lower limbs, positive Gower’s sign, decreased lower limbs muscle strength, patellar and plantar reflexes absent bilaterally, slight pes equinovarus deformity, axonal deterioration affecting motor and sensory function mainly of lower limbs^81^; (g) Peripheral neuropathy, characteristic hair, and cerebellar dysfunction, variable bony deformities, cranial nerve involvement and intellectual disability^4^

### Mitochondrial sensory polyneuropathy subtypes

#### POLG-related polyneuropathy

20-year-old girl with identification of *POLG* variants [c.2243G>C (p.W784S)]; [c.3609_3612dupAACT] showed progressive sensory ataxia, intractable generalized epilepsy, magnetic resonance imaging (MRI)-confirmed cerebellum atrophy, and speech delays. Although the c.2243G>C (pW784S) is a variant of uncertain significance (VUS), clinical correlation and identification of a second pathogenic variant suggested that the *POLG* gene in this case is disease causing. The patient showed normal muscle strength but had nystagmus, ataxia, and dysarthria as well as absent reflexes and died at age 21 years due to liver failure.

#### HADHA-related mitochondrial trifunctional protein (MTP) deficiency

A 14-year-old female showed recurrent rhabdomyolysis, retinitis pigmentosa, mitral valve insufficiency, seizures with episodic weakness and gait abnormalities. The patient demonstrated tightened heel cords, decreased lower-extremity reflexes, absent sensory nerve action potentials (SNAPs). Pathogenic variants in *HADHA* gene [c.1528G>C (p.E510Q)]; [c.1620 + 2_162 + 0delTAAGG] were identified in this patient confirming molecular diagnosis.

A 5 year-old boy with history of early onset motor delay and walking difficulty first presented to pediatric neuromuscular clinics and electrodiagnostic (EDX) laboratory. He had bilateral hand tremors, waddling gait, and absent reflexes in lower extremities. EDX confirmed sensory polyneuropathy. Soon after, he was admitted to a cardiac intensive care unit with a diagnosis of severe cardiomyopathy with cardiac failure. Exome sequencing revealed a clinically correlated disease-causing variant in *HADHA* gene [(c.1418C>A (p.Ala473Asp)] in this patient, and after cardiac transplantation, motor strength in lower extremities improved. Genetic testing on its own was not strictly confirmatory, as a second variant in the *HADHA* gene was not identified; however, clinical correlation of genetic testing results suggested *HADHA* as the most likely disease-causing gene.

#### COX20-related polyneuropathy

An 18-year-old girl with sensory neuropathy, ataxia, distal extremity weakness, spasticity, and hearing loss presented farsightedness and esotropia at 3 years of age and developed gait instability by age four. The patient also presented chronic gastrointestinal problems with a strong family history of biopsy-confirmed Celiac disease on the maternal side with negative results in brain and spine MRI, Charcot-Marie-Tooth and Friedreich ataxia-specific genetic-testing. Her 10-year-old younger brother, delivered as twin B at 36 weeks of gestation with a brief respiratory distress showed speech and gross motor delays. The patient sat up at 12 months, walked at 18 months and could run by age four at which the onset of alternating esotropia, strabismus, and gait ataxia were noted. At 5 years of age, hypotonia and hyperreflexia were recorded in this patient and subsequent EDX revealed sensory polyneuropathy. Whole exome sequencing (WES) revealed pathogenic variants in the *COX20* gene for both siblings: [c.157 + 3G>C (IVS2 + 3G>C)]; [c.41A>G (p.Lys14Arg)]; and [c.340G>A (p.Gly114Ser)]. c.157 + 3G>C variant was paternally inherited, and the p.Lys14Arg and p.Gly114Ser variants were maternally inherited.

#### FH-related fumarase deficiency

A 10-month-old boy with fumarate hydratase deficiency presented with global developmental delays, seizures, low muscle tone and absent tendon reflexes on neurological examination. His work up showed absent SNAPs on NCS, abnormal electroencephalogram (EEG) and cerebral atrophy on the brain MRI. The patient also had strabismus with some visual impairments, fatigue and poor weight gain, and anemia. In addition, some cardiac abnormalities such as pulmonary artery hypertension, and pulmonary stenosis, as well as urinary tract infection were also present. The patient harbored *FH* gene variants: [c.697C>T (p.R233C)]; [c.1431_1433dup].

### Spinocerebellar ataxia (SCA)

#### ATXN2-related spinocerebellar ataxia type 2

Identical 17-year-old twin brothers showed progressive ataxia, myoclonic jerks, feeding difficulty, and weight loss with an onset age of 10 years. The brothers also showed bradykinesia, spasticity, decreased pinprick, vibration, and feet proprioception, as well as dysmetria, absent reflexes in lower extremities and wide-based ataxic gait. Symptoms worsened with need for gastrostomy tube, anti-seizure medications and benzodiazepines. Brain MRIs showed cerebellar atrophy and NCS had absent SNAPs for both brothers. The older twin had genetic confirmation and harbored the pathogenic variant [c.1564_1565delGA (p.Glu522Ilefs43)] in *ATXN2*.

A 14-year-old girl with ataxia, dysphagia, and developmental delay with onset age of 4 years demonstrated dysarthria, increased tone in extremities and axial hypotonia, decreased vibration and proprioception, ataxia and dysmetria, and absent tendon reflexes. MRI brain showed diffuse cerebral and cerebellar volume loss. She had absent SNAPs on NCS and her genetic testing confirmed predicted pathogenic variant in *ATXN2* gene.

### Ataxia telangiectasia

#### ATM-related ataxia telangiectasia

Sisters aged 11 and 12 years at the time of examination with a single *ATM* gene pathogenic variant [c.1564_1565delGA (p.Glu522Ilefs43)] showed progressive ataxia, myoclonus, bulbar conjunctiva and skin telangiectasia, immunodeficiency on chronic intravenous immunoglobulins and recurrent skin infections. Even though a second variant was not identified, clinical correlation of genetic testing suggested the *ATM* gene was disease causing. On examination, patients demonstrated nystagmus, dysarthria, ataxia, absent tendon reflexes and absent SNAPs.

A 3-year-old female with normal early development with rolling and sitting at age 3 and 6 months respectively but later developing balance problems, drooling and fine motor problems and in-toeing of gait, was found to harbor a heterozygous pathogenic variant in *ATM* gene [c.4544dup (p.Asn1515Lysfs*16)], in addition to two VUSs, [c.7397C>A (p.Ala2466Glu)] and [c.7502A>G (p.Asn2501Ser)]. No seizures, headaches or focal weakness were noted in this patient, but frequent ear infections and difficulty in maintaining sleep were noted. No family history of neuromuscular or balance problems was found. The genetic testing results, taken together with the clinical suspicion for the disease, were suggestive of a diagnosis of Ataxia Telangiectasia.

### Friedreich's ataxia

#### FXN-related Friedreich ataxia

A 16 year old with confirmed genetic diagnosis of 899 GAA-repeats of *FXN* showed progressive gait instability, scoliosis, and absent SNAPs. The patient presented with good muscle strength but needed walking assistance with decreased vibration in her extremities with dysmetria, absent tendon reflexes and bilateral Babinski.

Three other children were evaluated with similar presentation including cardiomyopathy and attention deficit hyperactivity disorder (ADHD) with 800–1250 range *FXN* GAA triplet repeats among whom one patient also harbored one pathogenic missense variant [c.317 T>C (p.Leu106Ser)] in *FXN* gene.

### Spastic paraplegia (SPG)

#### ZFYVE26-related spastic paraplegia

A 16-year-old girl showed progressive ataxia, seizures, scoliosis, diffuse cerebellar atrophy, and hearing loss. This patient showed low muscle tone, absent reflexes in lower extremities, ataxia, and absent SNAPs. The patient harbored VUSs in *ZFYVE26* gene [c.2300G>A (p.R767H)]; [c.2799C>T (p.L933 =)] that are likely disease-causing variants in *ZFYVE26* based on clinical correlation suggesting SPG15 (OMIM# 612,012).

#### SPG7-related spastic paraplegia

A 17-year-old boy with scoliosis and gait abnormalities showed decreased lower extremities strength and reflexes with contractures, decreased vibration and temperature sensation. EDX showed absent SNAPs. MRI of brain/spine showed significant scoliosis. Genetic testing pointed towards *SPG7* gene with a likely pathogenic variant [exon 1: c.1A>G] with a possible autosomal dominant inheritance confirming diagnosis of SPG7 (OMIM# 602,783) in correlation with clinical data.

### Giant axonal neuropathy

#### GAN-related giant axonal neuropathy-1

A 6-year-old boy showed progressive gait abnormality, hammer toes, high arched feet bilaterally with 4/5 strength (MRC scale) in distal feet muscles, absent distal tendon reflexes in the lower extremities and intact in upper extremities. EMG findings were indicative of inherited moderate to severe sensory predominant polyneuropathy. MRI of brain showed evidence of symmetric signal abnormalities primarily involving the dentate nuclei of the cerebellum, deep cerebellar and cerebral white matter. Diminished N-acetylaspartate peak in MR spectroscopy suggested nonspecific neuronal dysfunction appearance but possible metabolic disorder such as a mitochondrial abnormality or an aminoaciduria among others. The patient was found to harbor a homozygous pathogenic variant [c.851 + 1G>A] in the *GAN* gene. Taken together, the clinical features, genetic results, and correlation with published evidence^4^ confirmed a giant axonal neuropathy-1.

Another 10-year-old girl harboring two pathogenic variants in a compound heterozygous manner in the *GAN* gene [c.805C>T (p.Arg269Trp)]; [c.732delT (p.Ile244MetfsX33)] showed wide based, high steppage gait with sensory ataxia with positive Rhomberg’s signs. The wide-based gait was worse with eyes closed or in the dark. Truncal ataxia was greater than appendicular ataxia. The subject showed decreased muscle bulk in the bilateral lower extremities and distal hands without any scapular winging and fast disease progression towards lower limb immobilization. Sensory evaluation revealed decreased vibration in joint position on pin prick in bilateral feet and absent deep tendon reflexes and flexor plantar responses. The patient also showed vocal cord paralysis, episodes of tachycardia, difficulty breathing, poor cough reflex, and difficulty in swallowing. Taken together, definitive molecular diagnosis confirmed giant axonal neuropathy-1.

## Discussion

In our study, we retrospectively analyzed pure sensory polyneuropathy cases of genetic origin seen over a period of 7 years at a tertiary care Children’s hospital electrodiagnostic laboratory (Table 1) and performed an English literature search for each gene-specific disease type (Table 2). Although limited by the heterogeneity of different sequencing tests performed at physician discretion, thorough analyses of a single-center experience of childhood genetic sensory polyneuropathy are needed to understand the etiologies and variabilities of each disease subtype.

In our study, sensory polyneuropathy of genetic origin accounted for less than 2% of all pediatric electrodiagnostics performed and affected young children without gender preference. Average delay to genetic confirmation was 5 years. All children showed gait problems secondary to large fiber predominant sensory polyneuropathy with loss of joint position and positive Rhomberg, spasticity with or without cerebellar component and varying degrees of learning difficulties, scoliosis, epilepsy, swallowing difficulty, hearing loss and cardiomyopathy as co-morbidities. All study subjects had no response in SNAP’s. Therefore, we were unable to assess and quantify disease severity variabilities based on nerve conduction velocity (NCV) in NCS. However, on clinical examination the severity of neurodegeneration varied. In all subjects, we identified length-dependent abnormality with absence of sensory modalities like reduced vibration sense, joint position and reduced or absent tendon reflexes to be present in bilateral distal lower extremities more than the upper extremities. Clinically, however, on NCS both upper and lower extremities showed no response.

Mitochondrial genes were the most common genetic etiology in our cohort. Up to 1/3^rd^ of mitochondrial disease patients may have a peripheral neuropathy but are often undiagnosed due to the severity of other symptoms^82^. In this report, we describe siblings with a novel *COX20*-associated disorder. *COX20* codes for a protein that is involved in the assembly and stability of mitochondrial complex IV, the final component of the respiratory chain. Clinical features include childhood-onset progressive cerebellar ataxia, sensory neuropathy, hypotonia, areflexia, dysarthria. The natural history of this condition is emerging recently albeit from small number of cases^27–30,83^. Interestingly, the two siblings even with similar genotypes showed some heterogeneity in clinical presentations, such as only one of them showing initial respiratory distress, which agrees with the established mechanism of respiratory chain assembly intermediate accumulation causing reduced respiratory capacity in the absence of COX20 protein as shown in HEK293 cells^84^. Thus, it is possible that other genetic modifiers not identified in this study could drive the phenotypic differences. We also did not observe the severe cognitive or intellectual disabilities of COX20-related diseases typically reported in literature. We also identified a *POLG*-associated female patient showing ataxia, seizures, speech and motor delays, ophthalmic abnormalities, and ultimately succumbing to liver problems at age 20 years, a feature seen in *POLG*-associated Alper-Huttenlocher syndrome in neonates or children^85^. Two unrelated patients in our cohort were diagnosed with mitochondrial trifunctional protein (*HADHA*) deficiency. The majority of neuropathy cases described includes sensorimotor polyneuropathy with pure sensory neuropathy being a less represented subset of *HADHA*-associated neuropathies^86–88^. Their symptoms correlated well with that of long-chain 3-hydroxyl-CoA dehydrogenase deficiency (LCHADD) characterized by early-onset cardiomyopathy, hypoglycemia, neuropathy, pigmentary retinopathy, and sudden death—although the patients in our cohort survived. Previously, it was suggested that children born of a mother with severe fatty liver pregnancy should be genetically tested for LCHAD or MTP^89^. Interestingly, the patient with two identified *HADHA* variants showed cardiac aortic valve insufficiency but not cardiomyopathy per se as in patients with one identified *HADHA* variant. Moreover, the two *HADHA* patients show clinical heterogeneity that needs to be accounted for, such as patient# 2 who showed rhabdomyolysis, ophthalmic defects and seizures unlike patient# 3. Further testing on patient# 3 such as genome sequencing to identify possible deletion or duplication or deep-intronic variant that exome sequencing may not find needs to be done to identify the second *HADHA* variant. We also identified a fumarate hydratase (fumarase) deficiency case which showed severe weakness, generalized severe hypotonia, focal epilepsy and infantile spasms and developmental delay. Fumarase is an enzyme involved in the mitochondrial Kreb’s cycle, converting fumarate to malate and hence this subtype should be included in the mitochondrial sensory polyneuropathy group as it causes generalized muscle hypotonic and brain and developmental disorders. Interestingly, the patient showed cardiac symptoms such as pulmonary stenosis and pulmonary artery hypertension, and urinary tract infection which are generally not observed in fumarase deficient children, suggesting broader clinical spectrum of cardiac comorbidities.

Sensory polyneuropathy is well-described Friedreich ataxias (FA) feature^90^ although the precise pathogenesis is controversial and considered secondary to mitochondrial metabolism dysfunction^91^. We identified four FA cases with patients# 14 and 16 harboring the largest *FXN* trinucleotide repeat length and showing more severe phenotypes including cardiomyopathy, scoliosis, bladder dysfunction and developmental delay as was previously suggested^61,64^. It is also interesting to note that patient# 14, with a single allele repeat and a missense variant, showed slightly milder phenotypes, similar to previous findings^66–68^. Peripheral neuropathy is a predominating symptom in spinocerebellar ataxia 2 (SCA2) patients with>80% having abnormal nerve function. Spinocerebellar ataxias may range from motor or sensory neuropathies to a combination of both. Other studies have demonstrated pure sensory neuropathy being most prevalent among SCA3^92^, but it was absent from our cohort. Ataxia telangiectasia is an autosomal recessive disorder affecting the neuromuscular- and the immune-system characterized by progressive cerebellar ataxia, oculomotor apraxia, choreoathetosis, oculocutaneous telangiectasia, frequent infections, and increased cancer risk with hematological malignancy being the most common^93^. Spastic paraplegias are a heterogeneous group of slowly progressive motor neuron disorders which presents frequently with lower limb spasticity and weakness^94^. Moreover, the SCA2 siblings age of disease onset and diagnosis were at 3 weeks which is, to our knowledge, the youngest cases reported so far being even younger than previously identified youngest case at 3 months^95^. Unlike reported symptoms in literature, the two siblings harboring *ATXN2* pathogenic variants show signs of epilepsy and anti-seizure medication had to be given (patients 7 and 8 in Table 1). It is likely that ATXN2 aberration causes a brain developmental issue as reflected in the developmental delay in patient# 9 (Table 1) as well.

We identified two cases of spastic paraplegia (SPG), specifically SPG15 (*ZFYVE26*) and SPG7 (*SPG7*), with SPG15 showing a bigger and broader phenotypic onslaught compared to SPG7 in the form of seizures, hearing loss, and cerebellar atrophy. Among the giant axonal neuropathy (GAN) cases, we identified uncommon features not found in the literature survey, such as tachycardia and breathing difficulty in particular for patient# 20, making the clinical presentation of GAN broader.

Overall, pathophysiological follow up of childhood sensory polyneuropathies are critical as it could potentially be a marker for later onset neurodegenerative diseases. Our study shows the importance of peripheral nerve degeneration in sensory ataxia and peripheral nerve involvement in rare genetic polyneuropathies. Electrodiagnostic findings of peripheral sensory polyneuropathy in the appropriate clinical context mandates further genetic testing and subsequent clinical correlation. A genetic diagnosis is important as it provides a unifying diagnosis to coordinate care, prognostic and anticipatory guidance for clinicians (cardiology referral for suspect mitochondrial and Friedreich ataxia patients) and families (breast cancer risk in *ATM* carrier mothers), recurrence risks for family planning purposes, and possible additional treatment options.

## Patients and methods

A parent or a legal guardian of all pediatric participants in this study provided written informed consent and the study was approved by the Children’s Healthcare of Atlanta (CHOA) and Emory University (EU) Ethics Committee and Institutional Review Board (CHOA IRB 13-151 and EU IRB00075815). All methods were carried out in accordance with relevant IRB guidelines and regulations. A retrospective chart review of the pediatric electrodiagnostic database at a tertiary care Children’s Hospital from 2013 to 2019 was performed. Children with absent sensory nerve action potentials (SNAPs) on nerve conduction studies (NCS) with remaining of the study being unremarkable were included. Clinical neuromuscular examination findings, genetic testing results and the clinical course of the above mentioned subjects were reviewed.

Board-certified pediatric neurologists performed the clinical examination and electrodiagnostic studies. Clinical examination focused on gait, motor strength, tendon reflexes, sensory modalities (pinprick, joint position, vibration and temperature) and Babinski and Romberg signs. SNAPs were recorded through antidromic electrode stimulation of sural, superficial fibular and plantar nerves using 15 mm disposable surface disc electrodes (part No. 019-415200; Natus Neurology, Middleton, WI) and on median and ulnar nerves (digits II and IV, respectively) using standard ring electrodes (part No. 9013S0332; Natus Neurology, Middleton, WI). Limb temperatures were kept between 28 and 32 °C and all data was collected using the Synergy software program (Natus Medical Incorporated, San Carlos, CA).

Genetic testing requests included *PMP* 22 deletion/ duplication, Friedreich ataxia trinucleotide repeats, spinocerebellar ataxia and whole exome sequencing (WES) or hereditary neuropathy gene-panel sequencing for patients with pure sensory polyneuropathy. Genetically confirmed cases (based on pathogenic variant identification and/or clinical correlation of the identified variants) were reviewed and variant pathogenicity was determined following American College of Medical Genetics and Genomics (ACMG) guidelines^96^. Disease-causing genes in cases of identification of variants of uncertain significance (VUSs) were determined by interpretation of most likely genetic cause based on clinical data correlation with genotype. Thereafter, an attempt was made to search and review similar published English literature cases.

### Ethics standards

All informed consents were obtained and the study was performed according to Institutional Review Board protocol. We, the authors, confirm that we have read the Journal's position on issues involved in ethical publication and affirm that this report is consistent with those guidelines.

## Data Availability

De-identified datasets will be available to other researchers from corresponding authors on reasonable request after publication as per Institutional Review Board protocol.

## Abbreviations

EDX: Electrodiagnostic
SNAPs: Sensory nerve action potentials
OMIM: Online Mendelian inheritance of man (https://www.omim.org/)
*COX20*: Cytochrome C oxidase assembly factor
*POLG*: Polymerase gamma
*HADHA*: Hydroxyacyl-CoA dehydrogenase/3-ketoacyl-CoA thiolase/enoyl-CoA hydratase
*FXN*: Frataxin
*ATXN2*: Ataxin-2
*ATM*: Ataxia-telangiectasia mutated
*ZFYVE26*: Zinc finger FYVE-type containing 26
*FH*: Fumarate hydratase
SPG: Spastic paraplegia
SCA: Spinocerebellar ataxia
GAN: Giant axonal neuropathy
